# The pro-neurotrophin receptor sortilin is a major neuronal APOE receptor for catabolism of amyloid-β peptide in the brain

**DOI:** 10.1186/1750-1326-8-S1-P10

**Published:** 2013-09-13

**Authors:** Anne-Sophie Carlo, Camilla Gustafsen, Guido Mastrobuoni, Morten Nielsen, Stefan Kempa, Claus Munck Petersen, Thomas Willnow

**Affiliations:** 1 Max-Delbrueck-Center for Molecular Medicine, Berlin, Germany; 2 MIND, Dept of Medical Biochemistry, Aarhus University, Aarhus, Denmark

## 

Sortilin is a member of the VPS10P domain receptor gene family, a class of sorting and signalling receptors in neurons. This gene family also includes SORLA/LR11, the neuronal sorting factor for APP. Sortilin has been shown to constitute an essential component of the receptor complex that transmits pro-neurotrophin-dependent death signals in neurons. In previous studies, sortilin-dependent pro-neurotrophin signalling has been implicated in regulation of neuronal viability during normal development and aging. Also, sortilin activity has been shown to control neuronal cell death and survival in spinal cord injury. Remarkably, up-regulation of pro-neurotrophins has been observed under conditions of neurodegeneration. These findings led us to hypothesize that sortilin signaling may not only control neuronal viability during acute but also during chronic distress of the nervous system as in Alzheimer’s disease (AD). Here, we have used mice with targeted sortilin gene disruption to address the consequences of impaired receptor activity for APP processing, structural and functional integrity of the brain, as well as AD pathology *in vivo.* When crossed with two different AD models (PDAPP, 5xFAD), sortilin-deficient mice showed a robust increase in Aβ levels in brain compared with control animals. Surprisingly, the levels of soluble APP products were not altered in sortilin-deficient mice, nor were the levels of Aβ-degrading enzymes, neprilysin and insulin-degrading enzyme, suggesting an impairment of Aβ clearance pathways. Apolipoprotein (APO) E is the major risk factor for sporadic Alzheimer disease. Among other functions, APOE is proposed to sequester neurotoxic amyloid-β peptides (Aβ) in the brain, delivering them to cellular catabolism via neuronal APOE receptors. In this study, we identified the pro-neurotrophin receptor sortilin as major endocytic pathway for clearance of apoliporotein E (APOE)/Aβ complexes in neurons. Sortilin binds APOE with high affinity. Lack of receptor expression in mice results in accumulation of APOE and of Aβ in the brain, and in aggravated plaque burden. Also, primary neurons lacking sortilin exhibit significantly impaired uptake of APOE/Aβ complexes despite proper expression of other APOE receptors. In spite of higher than normal brain APOE levels, sortilin-deficient animals display anomalies in brain lipid metabolism seen in APOE-deficient mice, indicating functional deficiency in cellular APOE uptake pathways. Taken together, our findings identified sortilin as an essential neuronal pathway for APOE-containing lipoproteins *in vivo* and suggest an intriguing link between Aβ catabolism and pro-neurotrophin signaling converging on this receptor.

